# Exploring the Lifestyle and Dietary Patterns of Food Supplement and Non-Food Supplement Users: A Cross-Sectional Study in the Portuguese Population

**DOI:** 10.3390/nu17172802

**Published:** 2025-08-28

**Authors:** Maria João Campos, Agnieszka Garbacz, Natalia Czlapka-Klapinska, Magdalena Czlapka-Matyasik, Angelina Pena

**Affiliations:** 1 Associated Laboratory for Green Chemistry (LAQV) of the Network of Chemistry and Technology (REQUIMTE), Laboratory of Bromatology, Pharmacognosy and Analytical Sciences, Faculty of Pharmacy, University of Coimbra, 3000-548 Coimbra, Portugal; mcampos@ff.uc.pt; 2 Department of Human Nutrition and Dietetics, Poznan University of Life Sciences, 60-624 Poznan, Poland; agnieszka.garbacz@up.poznan.pl (A.G.); czlapka.klapinska@gmail.com (N.C.-K.)

**Keywords:** food supplements, dietary patterns, cross-sectional study, cluster analysis, Portugal

## Abstract

**Background:** The increasing use of food supplements (FSs) and the knowledge gaps among healthcare professionals (HPs) and non-healthcare professionals (nHPs) in Portugal regarding their influence on dietary patterns and health need investigating. This study aimed to explore FS users’ lifestyle and dietary patterns, identifying differences and how professional background influences these patterns. **Methods:** A cross-sectional study was conducted among 1122 Portuguese adults aged 35 ± 14.0 yrs (between 18 and 85), via snowball sampling, collecting data on sociodemographic characteristics, dietary patterns, FS use, and health attitudes. Cluster analysis (k-means) revealed four patterns: (1) professional supplement users with a healthy diet (PSHD), (2) professional non-supplement users with a less healthy diet (PnSLHD), (3) non-professional supplement users with a healthy diet (nPSHD), and (4) non-professional non-supplement users with a less healthy diet (nPnSLHD). Logistic regression assessed associations with lifestyle traits. **Results:** Significant sociodemographic differences existed between HPs and nHPs. Higher nutritional knowledge and nutritionist recommendations were strongly associated with a healthier diet and FS use (PSHD, nPSHD). Smoking was associated with less healthy patterns (PnSLHD, nPnSLHD). Among nHPs, males were significantly more likely to belong to the nPSHD group (OR: 1.61) compared to females (OR: 0.61). Distinct dietary and lifestyle patterns among Portuguese FS users and non-users vary by background. **Conclusions:** The findings suggest that FS users often maintain healthier lifestyles, indicating that FSs typically supplement rather than compensate for poor habits.

## 1. Introduction

In 2024, 42.3% of the Portuguese population aged 16 and over reported having a chronic illness or long-term health problem [1,2]. Simultaneously, consumers increasingly seek alternative strategies to support their health and well-being, including using food supplements (FSs) [3]. The consumption of FSs has grown exponentially in recent decades, and it is expected to continue, raising questions about the actual need and potential risks associated with excessive consumption [4,5]. Their increased use is based on the perception that FSs contribute to a healthy lifestyle, eliminate dietary nutrient gaps, and help prevent diseases [6,7,8,9]. FSs should be considered a complement to a balanced diet, not a substitute for it. Nevertheless, their intended use may extend beyond this area: to complement deficiencies, assist treatment, or support special nutritional needs. Therefore, their use and recommendations should be discussed with health professionals.

Scientific evidence worldwide consistently shows that FSs are often used by subjects who are health conscious, eat a balanced diet, and have a high educational background [6,10,11,12]. Our previous studies highlighted a positive association between healthy lifestyle choices and FS consumption [13]. Participants with better eating habits and those with higher physical activity levels were more likely to use FSs, with significant differences between the factors influencing the use of FSs by healthcare professionals (HPs) and non-healthcare professionals (nHPs) [13].

Some European data indicate that one in five consumers has increased FS use over the past year, moving from a reactive, treatment-based approach to a more proactive, preventive mindset [14]. Data highlight that FS use is becoming an essential component of self-care, while a nutritious diet and regular exercise remain imperative strategies. A total of 29% of consumers take an FS for daily wellness [15,16]. It is crucial to recognise that the belief of many consumers that these products are safe is often unfounded [17]. The scientific literature shows that taking micronutrients without clinical justification does not lower the risks of cardiovascular disease and cancer, and that sometimes they can even have an adverse effect, which is pertinent in the context of chronic diseases [12,18,19]. There are even some opposite opinions about their use from some oncological experts [20,21,22]. Concerns about the safety of FSs, particularly when used unsupervised, have been noted by the scientific community.

It is crucial to underline that we have limited investigations on FSs, food consumption, and diseases, despite numerous studies worldwide investigating general patterns of FS use or dietary patterns as individual variables [15,23]. Some papers show a correlation between dietary choices or adherence to the Mediterranean Diet and FS consumption in sports populations [24,25,26]. There is a distinct lack of information regarding differences in dietary patterns and the specific factors influencing the use of FS among HPs versus nHPs, especially in the Portuguese context. To date, an optimal understanding of how a professional background and various sources of knowledge influence these choices has not been sufficiently established. Furthermore, there is a lack of data on analysing dietary patterns as a cluster of pro-healthy behaviours that distinguish FS users from non-users within these distinct professional groups. It is unclear whether the published research found a relationship between FS use, professional background, health, and dietary habits. This limited information provides the basis for further research on FS users’ dietary patterns and characteristics across different professional backgrounds.

Due to their professional knowledge, HPs play a vital role in guiding the use of supplements. Moreover, as authoritative figures, their practices and consumption patterns can influence public perception and recommendations. Factors driving FS consumption should be evaluated so we can understand how to advise and tailor supplements to the population’s needs [12,13]. As stated above, recent research has highlighted the nature of FS consumption. Socioeconomic factors, nutritional knowledge, and healthy lifestyles have all significantly influenced FS use [13]. However, despite the increasing focus on food safety as a research area, there remains a scarcity of studies examining the specific motivations and intentions driving FS consumers. The reasons for FS use are diverse. For many, it is based on the desire to enhance their overall health and wellness and address perceived nutritional gaps in their diets [9]. The need to boost the immune system seems to be a key motivator for older individuals [27]. Conversely, younger individuals often seek FSs to improve the health and condition of their skin and hair. Cultural and regional factors also play a significant role in the differences between countries [6,28]. Therefore, studies exploring the dietary patterns of HPs, FS users and those whose profession is not related to health sciences are needed. It would be crucial to find answers regarding the differences in dietary patterns and other traits that distinguish users from those who avoid FSs.

The prevalence of misinformation surrounding FSs adds to the complexity, leading to overconsumption and further compromising safety. Accurate FS consumption data are crucial to mitigate potential risks. To ensure the safe and rational use of FS, it is important to address these challenges through scientific research and effective regulatory measures. Several studies in the scientific literature focus on consumers’ perceptions of food safety and nutrition concerns [29]. It is essential to recognise the complex motivations behind FS consumption, ranging from addressing immediate health needs to mitigating perceived future risks. It would be imperative to study how food consumption patterns and their relationships with well-being affect the responsible use of FSs.

This study aims to fill this critical knowledge gap by investigating the lifestyle and dietary patterns of FS users and non-users within the Portuguese population. Specifically, this work seeks to identify differences in dietary patterns and pro-healthy habits between these two groups, with a focus on distinguishing healthcare professionals (HPs) from individuals in other professional backgrounds, referred to as non-healthcare professionals (nHPs). This study aims to answer the question of how FS users’ dietary patterns and lifestyle differ from those of non-FS users in the Portuguese population and whether these differences are influenced by professional background. This will contribute to defining the profile for FS recommendations for the Portuguese population.

## 2. Materials and Methods

### 2.1. Study Design

**Figure 1:**
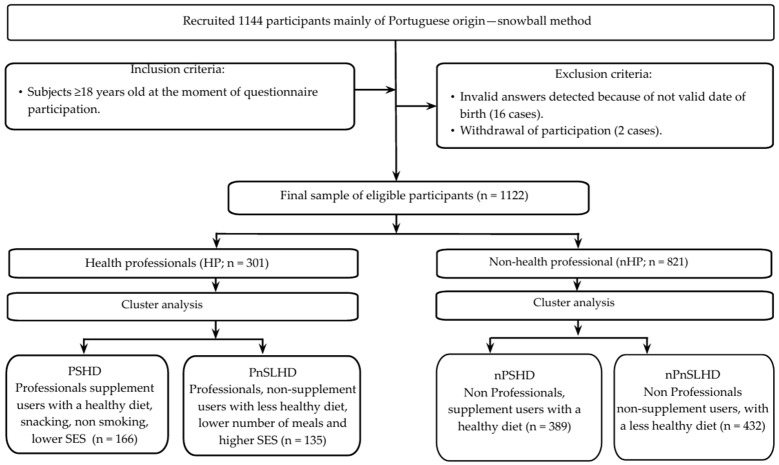
The subjects’ flowchart through this study.

This cross-sectional study was conducted among the adult Portuguese population, who were invited to participate by answering a questionnaire. A flowchart showing the subjects of this study is provided in Figure 1. Data were collected between 30 June and 30 September 2023. The snowball sampling method, a non-probabilistic sampling method, was used to enrol the study participants, who helped to spread the word and recruit additional respondents. The method was described previously [30]. First, this study was published on the authors’ personal and professional social media channels (i.e., LinkedIn^®^, Facebook^®^, and Instagram^®^), and requests were sent through discussion forums, professional organisations, mailing lists of students and teachers, and human resources departments of several national companies. The importance of sharing was reinforced through all requests on participants’ personal and professional social networks, as well as on WhatsApp^®^ groups. In this way, the chance of reaching a more diverse population increased. The investigators extended invitations to eligible participants who expressed interest in participating in this study, allowing them to complete an online, self-administered questionnaire.

A total of 1144 respondents voluntarily participated in this study. The inclusion criteria were being Portuguese, aged 18 years or older, and providing informed consent. Those who met these criteria were eligible to answer the questionnaire. Twenty-two participants were excluded because they did not meet the age requirement: four were under 18 when completing the questionnaire, and sixteen reported an invalid date of birth. Two additional participants indicated that they had no interest in answering the questionnaire. The characteristics of the collected study sample are presented in Table 1.

The Faculty of Medicine of the University of Coimbra Ethics Commission approved this study in June 2023 (process number CE-066/2023).

The final study sample was stratified a priori into two groups: (1) healthcare professionals (HPs) and non-healthcare professionals (nHPs). Health professionals included nutritionists and dietitians, pharmacists, medical doctors, dentists, nurses, veterinary doctors, physiotherapists, diagnostic and therapeutic technicians, and other professional therapies (e.g., acupuncture and osteopath) based on the question “What is your profession?”. Statistics were performed separately in both groups.

The qualification for supplement users and non-users was performed simultaneously and included in the statistical analyses. Food supplement users were defined as those who declared the use of food supplements in the 12 months preceding completion of the questionnaire. Non-food supplement users were those who denied using supplements in the last 12 months.

### 2.2. Data Collection Tools

An online self-administered questionnaire (Google^®^ Forms) was developed from the validated KomPAN^®^ dietary habits and nutrition beliefs questionnaire and other relevant published questionnaires [31,32,33,34,35]. The KomPAN^®^ consists of four sections: (1) dietary habits, (2) frequency of food consumption, (3) views on food supplements and nutrition, and (4) lifestyle and personal information [35]. All consumption frequencies that were collected in the questionnaire were evaluated in six categories (from “never” (1) to “a few times a day” (6)). For each food item, the categories of frequency of consumption were converted to values reflecting daily consumption frequency (the range 0–2 times/day) [32].

The final questionnaire of this study was piloted and pretested among 67 individuals. The questionnaire includes sociodemographic information, dietary habits, and participants’ knowledge, awareness, and attitudes related to health, particularly FSs. Questions from the developed survey were translated into Portuguese. All answers considered for this study were based on closed responses to reduce confounding factors and errors during interpretation. The questionnaire took an average of 25 min to complete.

### 2.3. Sociodemographic Information

The questionnaire included sociodemographic characteristics such as gender, date of birth, weight, height, nationality, education level, place of residence, number of subjects living in a house (including children), perception of financial situation, quality of life and profession. Based on the collected data, socioeconomic status (SES), a significant determinant of many behaviours, was calculated based on the literature [36,37]. The SES index was calculated as the product of the numerical values assigned to the individual categories of each SES factor, as described in detail in previous articles [13]. The more favourable categories of SES factors were assigned higher numerical values. After that, the SES was logarithmically transformed, and the tertiles of the SES distribution were used to categorise respondents into those with low, average, or high SES.

### 2.4. Dietary Habits Information

Diet quality indices were determined for the defined products and product groups—a pro-healthy diet index (pHDI-18), a non-healthy diet index (nHDI-27), a diet quality index (DQI) and a sweet diet index (SDI-13) [31]. The results were calculated based on the previously recommended division and interpreted according to the KomPAN^®^ protocol, with the adaptation to the Portuguese population. We used the nutritional and food group recommendations provided for the Portuguese population as a basis [33,38,39,40].

$$\sum Diet\;Indexes\left(pHDI-18;nHDI-27;DQI-,SDI-13\right)=\frac{100\times \sum A}{\sum B}(\%)$$

“A” represented the reported daily intake (times/day) for all items within specified food groups (pro-healthy, non-healthy, total, sugar sources). At the same time, “B” represents the sum of the maximum possible daily intake for those same foods, calculated for a single product as 2.

The pro-healthy diet index (pHDI) analysed the frequency of intake of 18 products/food groups: (1) water, (2) white bread, (3) dark bread, (4) white rice and pasta, (5) whole-grain pasta and rice, (6) milk, (7) fermented dairy, (8) fresh cheese curd product, (9) red meat, (10) white meat, (11) fish and shellfish, (12) eggs, (13) legumes, (14) potatoes, (15) fruits, (16) vegetables, (17) fruit juices and (18) vegetable juices or fruit and vegetable juices.

Non-healthy diet index (nHDI) analysed the frequency of intake of 27 products/food groups: (1) carbonated or non-carbonated drinks, (2) energy drinks, (3) fast food, (4) fried food, (5) butter, (6) lard, (7) vegetable oils, (8) cheese, (9) cured meats, (10) sweets, (11) instant soups, (12) canned meats, (13) canned vegetables, (14) candies, (15) cookies, (16) cakes, (17) chocolate bars, (18) muesli and granola bars, (19) donuts, (20) chocolate, (21) ice creams, (22) sweetened yogurts, (23) cakes and pastries, (24) gums, (25) jelly, (26) honey and (27) alcoholic beverages.

The sweet diet index (SDI) was created as an additional index to assess the intensity of dietary sources of sugars. The SDI analysed the frequency of intake of 13 products/food groups for frequency of consumption: (1) candy, (2) cookies, (3) cakes, (4) chocolate bar, (5) muesli/granola bars, (6) donuts, (7) chocolate, (8) ice cream, (9) sweetened yogurt, (10) cakes and pastries, (11) honey, (12) gums and (13) jelly.

The overall diet quality index (DQI) was calculated as the sum of all positive components of the pHDI and all negative components of the nHDI. In the calculations, weight scores (so-called weights) were used, which ensured the share of 18 components of the pHDI was the same as the share of 27 components of the nHDI. This index enabled a common interpretation of the consumption of foods with a potentially beneficial and negative influence on health by combining these opposing diet characteristics and expressing them in numerical values.

$$\sum DQI=\frac{100}{36}\times \sum C+(-\frac{100}{54}\times \sum D(\%)$$

“C” was the reported daily intake (times/day) for all items within specified pro-healthy food groups. “D” represented the sum of the daily frequency of intake for non-healthy foods.

The three dietary indexes: pHDI, nHDI and SDI were assessed for the KomPAN^®^ questionnaire across three intake intensities: low (0–33), medium (34–66) and high (37–100) based on the calculated percentage points [31]. The interpretation ranged between 0 and 100 points.

The range of the DQI was from −100 to 100 points. A higher value of the DQI indicates a higher quality of diet and a higher intensity of beneficial dietary characteristics for health. Based on the calculated points, the proposed interpretation of the DQI for the KomPAN^®^ questionnaire included three categories: high intensity of non-healthy dietary characteristics, presenting scores between −100 and −26, low intensity of unhealthy and pro-healthy traits with scores between −25 and 25 and high intensity of pro-healthy characteristics (26–100).

The indicators were modified in accordance with the number of product groups for this study, as in former studies [37]. The results were calculated based on the previously recommended division and interpreted according to the KomPAN^®^ protocol [31,38].

### 2.5. Attitudes Through Food Supplements and Health Information

The questionnaire collected information regarding participants’ knowledge, awareness, and attitudes toward health, particularly about FSs. Among the examined variables were: chronic diseases, chronic medication use, physical activity engagement, and smoking habits. Comprehensive data on FSs were also gathered, outlining their usage patterns, reasons for consumption, places of purchase, and sources of professional advice. Additionally, participants’ understanding of the definition of FSs was evaluated. Details about the data sources and methodology of these points are in Table S1. Supplementary data for variables concerning attitudes through FS and health information.

### 2.6. Data Analysis

After considering the confidence level (99%) and the margin of error (3.42%), the calculated minimum sample size was 624 subjects. Additionally, a separate calculation was performed using a pre-determined margin of error of 5% and a 27% response distribution for each question, given that we estimated we had 26,6% of FS users in Portugal, according to official data [40]. The required sample size was calculated using a 95% confidence level and a 5% margin of error, with a sample size calculator (www.calculator.net). This study’s minimum required sample size was 525, considering the adult population in Portugal (10,883,512) in 2023 [41,42].

All variables were checked for normality using the Kolmogorov–Smirnov test. The χ^2^ test was used to assess the distribution of the categorical variables. The study groups’ characteristics involved calculating percentages, frequencies, means, standard deviations, medians, and confidence intervals, which were set at 95%.

The four supplementation–diet–lifestyle patterns were derived through cluster analysis (k-means clustering was applied). Based on the results, four distinct subgroups were identified: within the health professionals and non-health professionals groups separately: (1) professional supplement users with a healthy diet (PSHD) and (2) professional non-supplement users with a less healthy diet (PnSLHD); and within the non-professionals: (1) non-professional supplement users with a healthy diet (nPSHD) and (2) non-professional non-supplement users with a less healthy diet (nPnSLHD). A logistic regression analysis was conducted to identify significant associations between the clusters created during the analysis and selected lifestyle traits. The dependent variable was cluster membership, specifically the groups identified among professionals (PSHD, PnSLHD) and non-professionals (nPSHD, nPnSLHD). Independent variables included sex, lifestyle factors such as physical activity and tobacco use, nutritional knowledge level and sources of FS use recommendations—factors that could influence the decision to use supplements. The results were adjusted for age and body mass index (BMI) to minimise the influence of confounding variables. Both unadjusted and adjusted data were presented to maintain transparency.

The calculations were performed using Statistica v.14.1 (StatSoft Polska sp. z o.o., Kraków, Poland) software.

## 3. Results

The study population comprised 1122 participants, of whom 301 (26.8%) were health professionals (HPs). Table 1 presents the general characteristics of the population and the two subpopulations (HP and nHP). Most participants were Portuguese (96.6%) and female (78.7%). No statistically significant difference was observed in gender distribution between the HP and nHP groups (*p* = 0.06). The average age of the total sample was 35 years; however, the HP group was older than the nHP (39 vs. 33 years, *p* < 0.001). The study sample presented an average BMI within the recommended range (23.3 kg/m^2^), with most respondents being of normal weight (68.8%). Residential locations varied, with 32.6% of participants living in villages. A statistically significant difference was found in the place of residence between the HP and nHP groups (*p* < 0.01). The number of housemates was predominantly 3–4 (62.3%), and this variable demonstrated a significant difference between the professional groups (*p* < 0.001). Most participants (62.8%) reported having no children in the household. Nevertheless, the presence of children in the household differed significantly between HP and nHP (*p* < 0.05). The analysis of socioeconomic status revealed that the HP group presented significantly higher SES (5.4 vs. 5.2; *p* < 0.001) than nHP.

Regarding educational status, 65.3% of all participants held a university degree. As expected, the education level significantly differed between HP and nHP groups (92.0 vs. 55%; *p* < 0.001). Most participants (87.9%) self-identified as having a middle-class financial situation, and this self-classification differed significantly between the HP and nHP groups (89.7 vs. 87.2%; *p* < 0.01). Self-reported quality of life was primarily “Normal” (46.1%) or “Relatively comfortable” (31.5%), regardless of the studied group. Full-time employment was reported by 56.6% of the participants, and employment status varied significantly between the HP and nHP groups (95.0 vs. 42.5%; *p* < 0.001). The prevalence of cigarette smoking in the total sample was 15.1%, with a significantly higher prevalence in the nHP group over the HP group (17.5 vs. 8.3%; *p* < 0.001).

**Figure 2:**
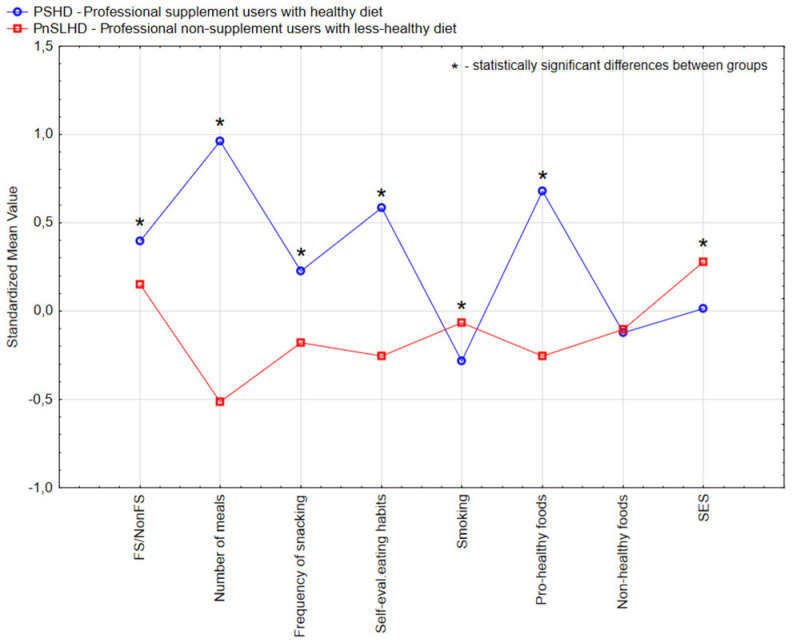
Two clusters’ visualisation of the standardised mean values characterising the health professionals group.

**Figure 3:**
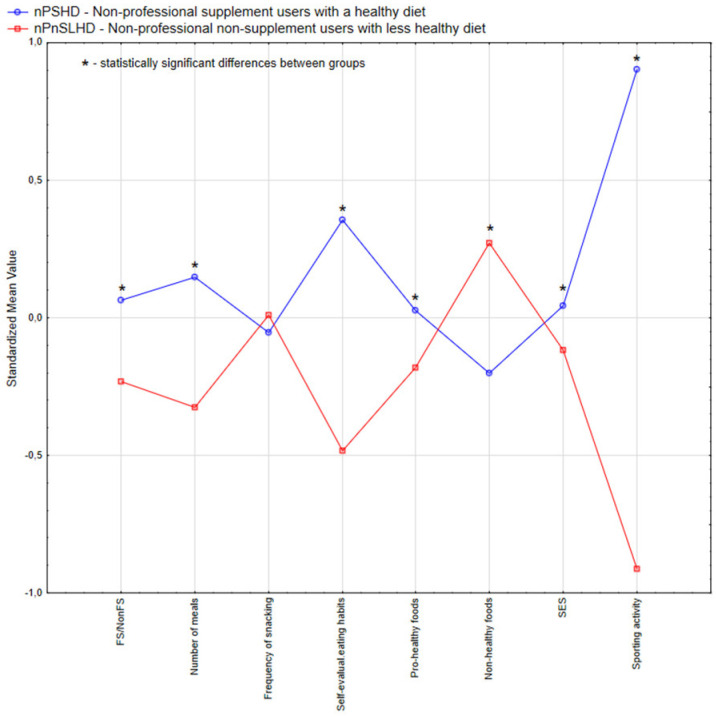
Two clusters’ visualisation of the standardised mean values characterising the non-health professionals group.

Using the information regarding profession (HPs or nHPs), intake of a FS over the last 12 months, the individual’s food consumption habits (number of meals per day, frequency of snacking between meals, self-evaluation of dietary habits, sum of pro-healthy and non-healthy foods intake), socioeconomic status (SES), attitudes toward sporting activity and smoking, we identified two clusters for each subpopulation, HP and nHP (Table 2 and Figure 2 and Figure 3). Detailed characteristics of the study sample by identified clusters are presented in Table A1.

The subpopulations HPs and nHPs were grouped into four distinct clusters. The HP subpopulation (Table 2 and Figure 2) was divided into the “PSHD—professional supplement users with a healthy diet” cluster (55.1% of the HP subsample) and the “PnSLHD—professional non-supplement users with a less healthy diet” cluster (44.9% of the HP subsample). The nHP subpopulation (Table 2 and Figure 3) was grouped into “nPSHD—non-professional supplement users with a healthy diet” and “nPnSLHD—non-professional non-supplement users with a less healthy diet” clusters, which comprised 47.4% and 52.6% of this population, respectively.

The cluster PSHD was characterised by FS consumers who used FSs (*p* < 0.05) in their daily routine, tended to eat more frequently at daily meals (*p* < 0.001) and snacks (*p* < 0.001). Their self-evaluation of eating habits indicated a healthier diet (*p* < 0.001); they consumed more pro-healthy foods (*p* < 0.001), smoked less (*p* < 0.001). 

The cluster “PnSLHD—professional non-supplement user with a less healthy diet”, represented HPs with the lowest (or lack) reported FS intake (*p* < 0.001), lower meal frequency (*p* < 0.001), lower self-evaluation of eating habits (*p* < 0.001); and lower pro-healthy foods intake (*p* < 0.05), higher non-healthy foods intake (*p* < 0.002), lower SES (*p* < 0.001), and declared lower engagement in sporting activity (*p* < 0.001).

The cluster “nPSHD—non-professional supplement user with a healthy diet” was characterised by individuals reporting higher FS consumption (*p* < 0.01) and eating more daily meals (*p* < 0.001). Their self-evaluation of eating habits indicated a more positive view of their diet quality (*p* < 0.001), reporting more ingestion of pro-healthy foods (*p* < 0.05), and fewer non-healthy foods (*p* < 0.002). This cluster also had a higher SES and reported greater participation in sporting activities (*p* < 0.001).

The cluster “nPnSLHD—non-professional non-supplement users with a less healthy diet”, representing nHPs with the lowest (or no) reported FS intake (*p* < 0.001), a lower number of meals a day (*p* < 0.001), a lower intake of pro-healthy foods (*p* < 0.05) and a greater intake of non-healthy foods (*p* < 0.002). In the nPnSLHD cluster, lower self-evaluation of dietary habits (*p* < 0.001), lower SES (*p* < 0.001), less frequent participation in sporting activities (*p* < 0.001) and more frequent smoking (*p* < 0.05) were noted.

To complement the graphical analysis of the clusters, the odds ratios (OR) for dietary lifestyle patterns related to the use of FS by selected factors in Portuguese adults are presented in Table 3. Gender influences diet and FS use patterns, primarily within the non-professional group in this Portuguese adult sample. Men were 1.6 times more likely to use FS, and women had a 40% lower likelihood of being FS users. Increased nutritional knowledge was consistently and strongly associated with a several-fold higher likelihood of being a supplement user in the HP group (PSHD, OR = 3.09 to 6.27). Conversely, higher nutritional knowledge is associated with a significantly lower likelihood of being in the PnSLHD group. For non-professionals, higher nutritional knowledge also predicts a higher likelihood of being nPSHD (ORs > 1) and a lower likelihood of being nPnSLHD (ORs < 1), all statistically significant. Therefore, nutritional knowledge strongly determines healthier diet patterns and FS use across HP and nHP groups. Healthy habits were also assessed. Smoking cigarettes is consistently associated with a lower likelihood of being PSHD or nPSHD. Smoking cigarettes increased the likelihood of being PnSLHD by almost three times and being nPnSLHD by one and a half times. Smoking seems to be a strong negative predictor for healthy diet and FS use, and a positive predictor for less healthy diets and no FS use. None of the HP recommendations showed statistical significance, except for the nutritionist recommendation, which is a powerful positive predictor for being PSHD (ORs > 3.75) and nPSHD (ORs > 3.13), with corresponding significantly lower likelihoods for PnSLHD and nPnSLHD. Recommendations from qualified health professionals (e.g., nutritionists) significantly drove healthier choices and FS use. The analysis highlights that nutritional knowledge, smoking habits, and nutritionist recommendations for FS significantly influenced dietary patterns and FS use across all groups. Gender differences are primarily observed within the non-professional cohort. The consistency of the ORs after adjustment for BMI and age suggests that these covariates did not substantially confound the observed relationships for most variables.

## 4. Discussion

In light of the significant prevalence and impact of chronic diseases on public health, understanding dietary patterns and the use of FSs becomes increasingly vital [1]. In Portugal, the prevalence of chronic illness or long-term health problems is high [2]. At the same time, according to market research, the percentage of the population regularly using FSs in Portugal has increased by 225% from 12.6% in 2011 to 26.8% in 2023, which aligns with global trends [43,44]. It is important to emphasise that FSs are not intended for disease prevention or therapy and should not replace a balanced diet, thus, discussing and reflecting on the relationship between FS use and dietary patterns is essential [45]. Therefore, in this study, we investigated the interrelationship between FS use, dietary patterns, and healthy lifestyle elements in Portugal.

### 4.1. FS Use and Dietary–Lifestyle Patterns

Our study contributes to understanding global trends in FS use by HPs and nHPs (the general population) and dietary patterns in Portugal, by drawing parallels with previous research studies from different countries [46,47,48,49,50,51,52,53]. For the HP clusters, high FS consumption (PSHD) is associated with a perceived healthier lifestyle, characterised by a better diet, higher meal and snack frequency, more pro-healthy foods, non-smoking status, and better sports habits, despite a lower SES. Conversely, HPs with low FS consumption (PnSLHD) tend to have a less healthy diet, a lower number of meals and snacking frequency, consume fewer pro-healthy foods, eat more non-healthy foods, have higher smoking rates, and less sporting activity, despite exhibiting a higher SES. For nHPs, the analysis also indicates a clustering of health-conscious behaviours and a slightly higher socioeconomic status among high-frequency users of FSs. FS use in the general population is often associated with better healthcare concerns. Those adults adopt healthy lifestyles and make informed, healthier dietary choices. Numerous studies have shown that using FSs correlates with higher SES and healthier lifestyles, including abstaining from smoking and engaging in regular physical activity [45,54,55].

Regarding the dietary aspect, several studies have shown a link between dietary patterns and the use of FSs [56]. As previously mentioned, the analysis of ORs reveals several significant factors related to an individual’s likelihood of belonging to specific clusters of dietary and lifestyle patterns and FS consumption. Our study has shown that higher nutritional knowledge is strongly and consistently associated with significantly increased odds of adopting a healthy diet and being placed in FS user clusters simultaneously (PSHD and nPSHD). The literature reveals several demographic and socioeconomic characteristics associated with supplement use, including higher education, better economic status, and healthy lifestyle [56]. A consistent trend emerges across HP and nHP populations in our study. Individuals with higher FS consumption report healthier dietary habits and more frequent meals, implying that FS users tend to perceive and maintain a healthier lifestyle, regardless of their professional background, which aligns with previous studies [13]. The perception of healthy food habits should be reinforced with a specific evaluation of food consumption categories. Table A1 reveals significant differences in various food consumption categories across the clusters, reflecting their “healthy diet” and “less healthy diet” labels. The more often consumed foods in the PSHD and nPSHD clusters were water, dark/whole-grain bread, whole-grain rice/pasta, fermented dairy products, fresh cheese, fish/shellfish, and eggs. These dietary choices resulted in significant differences between dietary scores calculated to define a priori dietary patterns in the study sample. Subjects from the PSHD and nPSHD clusters presented significantly higher DQI (diet quality intake) scores than those not using supplements. In the PSHD cluster, we noted that the DQI, referring to the overall diet quality, ranked at a high intensity of pro-healthy characteristics (29.5). In contrast, those without a health profession and not using supplements in their daily routine (nPnSLHD) had a DQI of 19.3, indicating a low intensity of unhealthy and pro-healthy dietary traits. The same group presented the highest score of the non-healthy diet index (nHDI, 8.8) among all clusters.

On the other hand, the least consumed foods in healthy diet groups (PSHD and nPSHD) are sugary and carbonated drinks, fast food, fried foods, butter and vegetable oils/margarines, processed cheese, sweets, canned food, and chocolate bars. Consequently, these same groups recorded the lowest SDI (6.0 and 5.2), indicating a low intake of sources of simple sugars in the diet.

These differences in dietary habits confirm that the clusters were well-formed based on diet quality. Individuals in the “healthy diet” groups consume more recommended foods and fewer processed items high in fats and sugars, confirming the validity of their classifications. The differences in specific food consumption habits provide valuable insights into the dietary choices that distinguish the groups. Individuals with healthier eating patterns, characterised by a higher intake of nutritious foods and lower intake of processed foods, also demonstrate greater use of FSs.

A positive relationship between dietary patterns and the use of FSs has been reported worldwide [46]. The use of FSs in the general population is commonly associated with health consciousness, which relates to factors such as adopting healthy lifestyles and making informed dietary choices. Numerous studies have demonstrated that using FSs is associated with higher SES and healthier lifestyles, including non-smoking and regular physical exercise and healthy dietary patterns [45,54,55]. Such observation is consistent with the “inverse supplement hypothesis”, which hypothesises that individuals who use FS are frequently those who need them less [47,48].

Studies from various countries suggest that FSs users tend to have healthier dietary habits [48,49,57]. They consume more fruits, vegetables, and fibre, while having a lower intake of fat [48,49,57]. Individuals consuming diets high in fat, low in fibre, or low in fruits are less likely to use FSs. Another study found that individuals with healthier lifestyles were more likely to use multivitamins and mineral supplements [9]. American FSs users with higher dietary intakes of essential vitamins and minerals (typically associated with fruits and vegetables) in their diets exceeded recommended micronutrient levels, in contrast to non-FS consumers [58]. Such data suggest a relationship between FS use and lifestyle choices in the US.

International studies from Switzerland, France, Denmark, Greece, and Germany consistently show that FSs users tend to have healthier dietary habits [50,51,52,53,59,60,61]. Specifically, they consume more fruits, vegetables, and whole grains, and less meat, fats, and sugary drinks. For example, a French study found that women with a healthy, plant-based diet were more likely to use FSs [59]. Additionally, a German study observed that FS users consumed more milk, dairy products, fish, and cereals [60]. This geographic overview corroborates the finding that the use of FSs is associated with a more balanced and health-conscious diet across European countries.

Contrary to European findings, China and Japan found no significant association between dietary patterns rich in fruits and vegetables and the use of FSs [46,62]. The survey in Japan even found that FS users had lower intakes of fruits, vegetables, and protein than non-users, indicating a tendency towards less healthy dietary patterns. These contrasting findings may reflect cultural differences in dietary habits and FS use between China, Japan and other European countries [63].

Ilowiecka et al. (2022) found evidence in an international study, involving 3500 FSs consumers, that FSs might be a substitute for a balanced micro- and macronutrient intake in adults with less healthy eating habits [64].

Healthier food choices, a higher level of education, increased physical activity, and a lower BMI were also identified as significant predictors of FSs intake [65,66]. Another frequently cited motivation for using FS is concern for overall health and well-being, and this habit is often linked to other health behaviours aimed at reducing potential health risks [67].

These results suggested a link between FSs use and healthier dietary choices, implying that FSs users are often more health conscious. They also reflect the diversity in dietary and cultural habits across countries, emphasising the importance of understanding the specific context in Portugal. Official nutrition guidelines are expressed in terms of foods, and a nutrient-oriented supplement does not guarantee the quality of the dietary pattern. Public health messages for health promotion do not advocate FSs to improve the population’s nutritional status [68].

### 4.2. FS Use and Healthy Attitudes

Other factors and respondents’ attitudes towards using FS in our study included smoking and sports participation. Smoking is consistently and significantly associated with decreased odds of being in healthy diet/FS user clusters (PSHD and nPSHD). Conversely, smoking substantially increases the odds of being in less healthy diet/non-FS user clusters (PnSLHD and nPnSLHD, which aligns with a French study [59]. In the nHP group, physical activity is a highly distinguishing factor, with 93.5% of supplement users with a healthy diet (nPSHD) reporting sports activity, compared to only 6.5% of non-supplement users with a less healthy diet (nPnSLHD). Sports activity is a decisive distinguishing factor for healthier lifestyle patterns among nHP [54]. In the nHP group, males were significantly more associated with a healthy diet and FSs use, while females were linked to a less healthy diet and no FS use. This finding is notable as it contrasts with general trends often observed in health behaviours in previous international studies, which reported that women predominantly consumed FSs [54,63,69]. This finding suggests that motivations for taking FSs vary among different population groups.

### 4.3. Pharmaceutical Perspective—FS Knowledge, Recommendation and Place of Purchase

Legally, FSs are concentrated sources of nutrients (i.e., minerals and vitamins) or other substances with a nutritional or physiological effect that are marketed in “dose” form (e.g., pills, tablets, capsules, liquids in measured doses) [70]. Most users assume FSs are safe because they resemble food and do not interfere with their therapeutic medications. Consequently, it is important to highlight the potential risk, as many FSs users are unaware of the potential for interactions between supplements and medications. Our results indicated that most respondents (approximately 80%), incorrectly stated the definition of FS, regardless of whether they use supplements or not, and their cluster affiliation. This outcome warrants discussion not only because the preferred point of purchase is the pharmacy, but also because the influence of the nutritionist’s recommendation on FS consumption is highly relevant.

A 2022 study by Chiba and Tanemura found that many FSs users are unaware of potential interactions between FSs and prescription medications. The study highlighted that only 30% of FSs users informed their doctors or pharmacists about FSs use, which poses a potential risk [28].

The use of FSs must be recommended by nutritionists, physicians and pharmacists, who should provide recommendations from a professional knowledge perspective. The aforementioned competences in HPs have been extensively discussed in studies worldwide, revealing diverse perspectives among professional groups and countries. Global surveys have shown that, despite HPs’ important role in advising on FSs, their understanding of FSs is limited and they lack confidence when providing consultation [71,72]. Doctors and pharmacists, with inadequate knowledge of FSs, often lack this understanding because education on this topic should be extended in the university curriculum [56]. Therefore, it may be advantageous to emphasise educating pharmacists on how to critically assess the utilisation, effectiveness, and safety of FSs (including identifying potential adverse effects and drug interactions), as well as how to engage with patients to address these and other issues related to FSs [72].

Findings by Strocka et al. (2024) indicate a relatively high level of awareness among healthcare professionals, a fact supported by scientific literature that shows a growing recognition of FSs among HPs [73]. These results are consistent with previous HPs’ surveys, revealing a lack of pharmacists’ knowledge and understanding of FSs [13,74]. Multiple surveys have indicated that pharmacist lack understanding and confidence regarding the safety and benefits of FSs and drug-FSs interactions [74,75,76]. Conversely, a study from Croatia found that while pharmacists scored well on a general knowledge test regarding FSs, their recommendations were often based on unreliable sources, like product labels, rather than safety and effectiveness data [77]. This finding highlights a gap between knowledge and practice among pharmacists, which can impact patient safety. The literature suggests that more training is needed for pharmacists on FSs, and further research should explore their experiences and educational background in this area [74]. Limited pharmacists’ knowledge and awareness of FSs has led to their expressed desire for increased FSs training [78,79]. Given that pharmacists regularly guide patients regarding FSs for various health conditions, they must possess comprehensive and up-to-date knowledge [73]. This finding becomes even more critical when our data reveal that pharmacies are the most commonly used place to buy FSs among the Portuguese.

Recommendations from healthcare professionals, such as nutritionists, are one of the most important factors leading to healthier dietary habits and FS use, among both healthcare professionals (HPs) and the general public. This result highlights the importance of nutritionists’ advice in using FS (in addition to pharmacists’ advice) for the entire population (including HPs), especially for those who already adopt healthier habits and use FSs. HPs are expected to have more health knowledge and access to varied sources of information; however, recommendations from another professional in the field for FS use seem to be a determining factor. It is also relevant that the PSHD group has a significantly higher proportion of nutritionists (28.9%) in its composition, underscoring the strong link between the profession and the behaviour of FS use with a healthy diet. What aligns with the study of Dickinson et al. (2012), which reports that dietitians, similar to other HPs, utilise FSs personally and recommend them to their patients [80]. The primary reasons for individual use of FSs and for endorsing them to clients were identical: promoting bone health, enhancing overall health and wellness, and addressing nutrient deficiencies in the diet [81]. Given the record levels of FSs use, HPs must understand their impact on patients’ health and maintain a fundamental knowledge base to communicate effectively with users of these products. The high use of FS leads to significant expenditure in the health system, and public health impacts must be considered. Community pharmacies, as an integral part of the healthcare system, provide essential health services that support health and significantly affect the outcomes of public health programs. They also have complementary and essential collaborations with nutritionists and medical doctors for the correct use of FSs. For the nHP group, there were differences between clusters in the place where FSs were purchased (*p* < 0.05), with the nPnSLHD group showing a tendency to acquire more from pharmacies compared to the nPSHD group. Although not significant for HPs in the presented data, pharmacies consistently serve as a relevant purchase location across all groups. This finding reinforces the role of pharmacies as a preferred channel and, for nHP individuals with less healthy habits who use supplements, as a trusted source for acquiring these products. Pharmacists may be essential in promoting healthier habits among their clients, as they are often regarded as a trusted source of health information [82]. According to the 2019 report of the Italian Centre for Social Studies and Policies (CENSIS), 95% of FSs are sold in pharmacies (86%) and in parapharmacies (9%) [83]. The same report confirms the key role of pharmacists in advising citizens on the purchase and use of these products, as 82% of Italians were advised on the purchase of FSs by either a doctor or a pharmacist [83]. However, a 2016 survey of Italian pharmacists found that 26% of respondents reported a lack of information on choosing the most appropriate product for the consumer, potential side effects, or interactions with other products [84]. An Australian survey found that 87–92% of consumers expected pharmacists to provide adequate and reliable information about the safety and benefits of FSs [85]. Most patients believe FSs are equivalent to food and do not interact with their medications. Patients’ preference for face-to-face consultations to discuss the use of FSs with medication underscores the importance for healthcare professionals, particularly pharmacists, to ensure the safe and appropriate use of FSs [86,87].

Many FSs users may use these products in conjunction with prescribed medications. For this reason, information about the interaction risk should be provided not only to patients taking medications but also to FSs users, either directly or indirectly, through healthcare professionals. Table 3 shows no statistically significant differences in “Presence of chronic illness” or “Intake of medications chronically” between the various HP and nHP clusters, or within their subgroups. Although an association between less healthy diets and a higher prevalence of chronic diseases or medication use might be expected, these results suggest that, for the studied sample, the presence of chronic illness or chronic medication use is not the distinguishing factor among the clusters defined by dietary patterns and FS use. This could mean that these factors are prevalent across the entire studied population, regardless of their diet or supplementation pattern, or that the impact of these factors on adherence to a healthy diet and FS use is more complex and might be mediated by other variables not directly captured by this table. Nevertheless, it remains a central topic of study that deserves further exploration in future studies.

Under current legislation, FSs are not classified as drugs. Therefore, their prescription or recommendation is not restricted to HPs, though the roles of pharmacists, physicians and nutritionists are essential for ensuring safe and rational use. Pharmacists have a unique position and accessibility to detect potential interactions and educate patients about the use, efficacy, side effects, and potential interactions with prescription medications associated with FSs. More awareness about FSs is needed so HPs can advise their patients. Some respondents in our survey started using FS for therapeutic purposes after receiving recommendations from HPs, which could benefit patients’ nutritional treatments. Better education about FSs is needed for HPs.

### 4.4. Limitations

It is necessary to note that the cross-sectional study design prevents causal inferences. Moreover, the online snowball sampling method may limit the generalisability to the wider adult Portuguese population. Nevertheless, this study has a substantial sample size of 1122 participants and minimal exclusion criteria, which minimises the potential for selection bias. Additionally, to address this, detailed sociodemographic characteristics of the entire study sample were provided, and differences between HP and nHP subpopulations were examined to provide a high-quality, comprehensive analysis. This approach improves comparisons with the limited number of Portuguese studies, highlighting concordant results while providing critical analyses of divergent findings.

Another limitation common to these studies is the self-reported nature of food frequency data, which introduces the possibility of reporting and recall bias. This study also has a disproportionate representation of women (79%) compared to the 2023 female adult Portuguese population data [88]. However, within the HP subpopulation, female representation (84%) aligns more closely with the percentage of women working in the Portuguese national health system in 2023, suggesting a more representative gender balance within the HP group [89]. The inherent limitations in the elderly’s access to technology, combined with the use of an online questionnaire, may have led to a non-random response pattern, with a lower participation rate in this age group.

Despite the limitations, we believe this study has several strengths, including the large number of participants, a validated international questionnaire, detailed characterisation of the study sample, and subpopulation analyses. Assessing the relationship between dietary patterns and the FS consumption is an innovative way of looking at this topic in the Portuguese population. This work provides valuable scientific insights into the existing literature.

## 5. Conclusions

Our research focused on analysing the dietary and lifestyle patterns of potential FSs users in Portugal. Our outcomes conclusively highlight the critical role of professional background in understanding FSs use and patterns associated with a healthier lifestyle among Portuguese adults, advocating for tailored public health strategies that utilise nutritional knowledge and professional guidance. The relatively large sample size allowed us to distinguish between subjects with healthcare-related backgrounds and those working in other professions. Our findings reinforce that FSs are typically integrated into existing healthier lifestyles as a supplement rather than used to compensate for poor habits or dietary deficiencies related to a health condition. The results demonstrate that FSs users generally exhibit healthier dietary and lifestyle patterns regardless of profession compared to non-FS users. Additionally, those with healthier dietary–lifestyle patterns reported higher nutritional knowledge, higher levels of sports activity and non-smoking status.

It is necessary to underline that nutritionist recommendations are associated with a more than three times increased likelihood to use supplements and follow healthy dietary and lifestyle patterns. A higher likelihood of using supplements was noted after the trainers’ recommendation in the non-health professionals. Surprisingly, this relationship was not observed after the pharmacists’ supervision. We postulate that this is most likely due to a lack of pharmaceutical counselling linked to the appropriate education of this professional group.

In light of our results, we emphasise a significant concern regarding the global consumption of FSs, considering factors such as who advises on them and who dispenses them. It is also important to consider the extent of knowledge about the recommended products and their effects in a given area, such as training, nutritional deficiencies, and the concomitant use of medications. This research’s significance lies in its novel contribution to improve knowledge on the relationship between FSs use, dietary habits, lifestyle choices, and professional background within the Portuguese context, a previously underexplored area. We are concerned about an alarming trend of FSs promotion aimed at sales outcomes rather than being tailored to particular consumer segments with specific needs. The importance of FSs for addressing nutritional gaps is evident, but we must also be aware of the associated risks. By recognising that professional background influences these dietary and lifestyle patterns, public health interventions can be designed to promote reasonable recommendations that correspond to the needs of specific population segments. Furthermore, a unified system for recommending FSs among all health professionals should be selected. We emphasise the importance of creating clear and unbiased guidelines for the advice and prescription of these products, which should be supported by strengthened studies on FSs in the curricula of nutritionists, physicians, and pharmacists. These guidelines should be global, reflecting the increasing movement of people. However, our primary concern is to start with a more rational and appropriate use of FSs in Portugal.

Future research should explore the mutual relationship between dietary patterns and potential nutritional deficiencies across all age groups. Furthermore, it is crucial to investigate the effectiveness of targeted educational programs and HPs’ recommendations in promoting healthier habits and responsible and adequate FSs consumption.

## Figures and Tables

**Table 1:** Characteristics of the study sample.

Variables	Total Sample	HP ^1^	nHP ^2^	*p*
Sample size *n* (%)	1122 (100.0)	301 (26.8)	821 (73.2)	
Gender				
Male *n* (%)	235 (20.9)	48 (16.0)	187 (22.8)	NS *p* = 0.06
Female *n* (%)	883 (78.7)	253 (84.0)	630 (76.7)	
Non-binary *n* (%)	3 (0.3)	0 (0.0)	3 (0.4)	
I do not want to report *n* (%)	1 (0.1)	0 (0.0)	1 (0.1)	
Age (years) Mean ± SD	35 ± 14.0 (18–85)	39 ± 11.0 (23–71)	33 ± 15.0 (18–85)	*p* < 0.001
(min–max)				
BMI ^3^ (kg/m^2^) Mean ± SD	23.3 ± 3.7	23.0 ± 3.7	23.3 ± 3.7	NS *p* = 0.16
(min–max)	(14.1–41.0)	(16.9–41.0)	(14.1–40.1)	
Underweight (<18.5)	55 (4.9)	11 (3.7)	44 (5.4)	NS *p* = 0.45
Normal (18.5–24.99)	772 (68.8)	217 (72.1)	555 (67.6)	
Overweight (≥25.0–30.0)	227 (20.2)	56 (18.6)	171 (20.8)	
Obese (≥30.0)	68 (6.1)	17 (5.6)	51 (6.2)	
Place of residence *n* (%)				
Village	366 (32.6)	68 (22.6)	298 (36.3)	*p* < 0.01 Chi^2^ = 8.5
City < 20,000 inhabitants	128 (11.4)	45 (15.0)	84 (10.2)	
City (20,000–100,000) inhabitants	302 (26.9)	85 (28.2)	216 (26.3)	
City > 100,000 inhabitants	326 (29.1)	103 (34.2)	223 (27.2)	
Number of housemates *n* (%)				
1–2	296 (26.4)	107 (35.6)	49 (6.0)	*p* < 0.001 Chi^2^ = 17.8
3–4	699 (62.3)	165 (54.8)	140 (17.1)	
≥5	127 (11.3)	29 (9.6)	97 (11.8)	
Children living in a house *n* (%)				
0	705 (62.8)	178 (59.1)	189 (23.0)	*p* < 0.05 Chi^2^ = 7.8
1–2	382 (34.0)	107 (35.5)	535 (65.2)	
≥3	35 (3.2)	16 (5.3)	97 (11.8)	
Status SES ^4^ Mean ± SD	5.2 ± 1.0	5.4 ± 0.9	5.2 ± 1.1	*p* < 0.001
(min–max)	(1.1–7.6)	(2.1–7.5)	(1.1–7.6)	
Status SES ^4^ (categorisation) *n* (%)				
Low	362 (32.3)	67 (22.3)	296 (36.1)	*p* < 0.001 Chi^2^ = 21.1
Average	398 (35.5)	128 (42.5)	269 (32.8)	
High	362 (32.3)	106 (35.2)	256 (31.2)	
Education level *n* (%)				
Primary school	64 (5.7)	4 (1.3)	60 (7.3)	*p* < 0.001 Chi^2^ = 156.2
High school	275 (24.5)	2 (0.7)	273 (33.3)	
University	733 (65.3)	277 (92.0)	456 (55.5)	
PhD	50 (4.5)	18 (6.0)	32 (3.9)	
Self-declared financial situation *n* (%)				
Low class	109 (9.7)	18 (6.0)	91 (11.1)	*p* < 0.01 Chi^2^ = 12.3
Middle class	986 (87.9)	270 (89.7)	716 (87.2)	
High class	27 (2.4)	13 (4.3)	14 (1.7)	
Self-declared quality of life *n* (%)				
Modestly poor	10 (0.9)	2 (0.7)	9 (1.1)	NS *p* = 0.21
Modestly	147 (13.1)	32 (10.6)	115 (14.0)	
Normally	517 (46.1)	133 (44.2)	382 (46.5)	
Relatively comfortably	353 (31.5)	103 (34.2)	251 (30.6)	
Comfortably	95 (8.5)	31 (10.3)	64 (7.8)	
Employment *n* (%)				
Full-time worker	635 (56.6)	286 (95.0)	349 (42.5)	*p* < 0.001 Chi ^2^ = 250.5
Temporary job	487 (43.4)	15 (5.0)	472 (57.5)	
Smoking cigarettes *n* (%)				
Yes	169 (15.1)	25 (8.3)	144 (17.5)	*p* < 0.001 Chi ^2^ = 14.7
No	953 (84.9)	276 (91.7)	677 (82.5)	
Nationality *n* (%)				
Portugal	1084 (96.6)	291 (96.7)	793 (96.6)	NS *p* = 0.16
Others	38 (3.4)	10 (3.3)	28 (3.4)	

^1^ HP—healthcare professionals; ^2^ nHP—non-healthcare professionals; ^3^ BMI—body mass index; ^4^ SES—socioeconomic status.

**Table 2:** Clusters characteristics by elements of the dietary–lifestyle patterns.

Variables	Total Sample	HP ^1^		*p*	Total Sample	nHP ^2^		*p*
	HP ^1^	PSHD ^3^	PnSLHD ^4^		nHP ^2^	nPSHD ^5^	nPnSLHD ^6^	
Sample size *n* (%)	301 (100.0)	166 (55.1)	135 (44.9)		821 (100.0)	389 (47.4)	432 (52.6)	
Food supplementation last 12 months *n* (%)								
Yes	182 (60.5)	110 (66.3)	72 (53.3)	*p* < 0.05 Chi^2^ = 5.2	344 (41.9)	193 (56.1)	151 (43.9)	*p* < 0.001 Chi^2^ = 18.1
No	119 (39.5)	56 (33.7)	63 (46.7)		477 (58.1)	196 (41.1)	281 (58.9)	
Dietary habits (Mean ± SD)								
Number of meals per day	3.9 ± 0.9	4.5 ± 0.6	3.1 ± 0.7	*p* < 0.001	3.5 ± 0.9	3.7 ± 0.9	3.3 ± 0.9	*p* < 0.001
Frequency of snacking between meals	1.0 ± 0.7	1.1 ± 0.8	0.8 ± 0.7	*p* < 0.001	0.9 ± 0.7	0.9 ± 0.7	1.0 ± 0.7	NS *p* = 0.42
Self-evaluation of dietary habits	3.2 ± 0.5	3.3 ± 0.5	2.9 ± 0.4	*p* < 0.001	3.0 ± 0.5	3.2 ± 0.5	2.8 ± 0.5	*p* < 0.001
Sum of pro-healthy foods intake	11.6 ± 3.6	13.8 ± 3.2	10.7 ± 2.8	*p* < 0.001	11.3 ± 3.6	11.6 ± 3.7	11.0 ± 3.5	*p* < 0.05
Sum of non-healthy foods intake	4.0 ± 2.7	3.6 ± 2.3	3.6 ± 2.7	NS	4.0 ± 2.7	3.3 ± 2.1	4.6 ± 3.1	*p* < 0.002
SES ^7^ (-) (Mean ± SD)	5.4 ± 0.9	5.4 ± 0.8	5.6 ± 0.9	NS	5.2 ± 1.1	5.3 ± 1.0	5.1 ± 1.1	*p* < 0.001
Declaration of sporting activity *n* (%)								
yes	176 (58.5)	100 (56.8)	76 (43.2)	NS Chi^2^ = 0.5	402 (49.0)	376 (93.5)	26 (6.5)	*p* < 0.001 Chi^2^ = 672.9
no	125 (41.5)	66 (52.8)	59 (47.2)		419 (51.0)	13 (3.1)	406 (96.9)	
Smoking currently *n* (%)								
yes	25 (8.3)	8 (4.8)	17 (13.0)	*p* < 0.05 Chi^2^ = 5.9	144 (17.5)	55 (38.2)	89 (61.8)	*p* < 0.05 Chi^2^ = 5.9
no	276 (91.7)	158 (95.2)	118 (87.0)		677 (82.5)	334 (49.3)	343 (50.7)	

^1^ HP—healthcare professionals; ^2^ nHP—non-healthcare professionals; ^3^ PSHD—professional supplement users with healthy diet; ^4^ PnSLHD—professional non-supplement users with less healthy diet; ^5^ nPSHD—non-professional supplement users with healthy diet; ^6^ nPnSLHD—non-professional non-supplement users with less healthy diet; ^7^ SES—socioeconomic status.

**Table 3:** Odds ratios (Ors) with 95% confidence intervals (CI95) of healthy diet and life patterns and use of food supplements by single factors in Portuguese adults.

Variables		PSHD ^1^, *n* = 166				PnSLHD ^2^, *n* = 135				nPSHD ^3^, *n* = 389				nPnSLHD ^4^, *n* = 432		
		OR (CI 95); *p*				OR (CI 95); *p*				OR (CI 95); *p*				OR (CI 95); *p*		
	*n* (%)	Without Adjustment	Adjusted for BMI ^5^	Adjusted for BMI ^5^ and Age	*n* (%)	Without Adjustment	Adjusted for BMI ^5^	Adjusted for BMI^5^ and Age	*n* (%)	Without Adjustment	Adjusted for BMI ^5^	Adjusted for ^5^ BMI and Age	*n* (%)	Without Adjustment	Adjusted for BMI ^5^	Adjusted for BMI ^5^ and Age
Insufficient nutritional knowledge level	5 (3)	-	-	-	5 (4)	-	-	-	18 (5)	0.26 (0.15; 0.44); *p* < 0.001	0.25 (0.15; 0.43); *p* < 0.001	0.25 (0.14; 0.43); *p* < 0.001	69 (16)	3.92 (2.29; 6.72); *p* < 0.001	3.96 (2.31; 6.80); *p* < 0.001	4.03 (2.34; 6.92); *p* < 0.001
Nutritional knowledge at insufficient or sufficient level	41 (25)	0.32 (0.20; 0.53); *p* < 0.001	0.30 (0.18; 0.50); *p* < 0.001	0.29 (0.17; 0.48); *p* < 0.001	68 (50)	3.09 (1.90; 5.05); *p* < 0.001	3.34 (2.01; 5.54); *p* < 0.001	3.46 (2.07; 5.77); *p* < 0.001	218 (56)	0.50 (0.38; 0.67); *p* < 0.001	0.51 (0.38; 0.68); *p* < 0.001	0.50 ‘(0.38; 0.68); *p* < 0.001	310 (72)	1.99 (1.49; 2.66); *p* < 0.001	1.96 (1.47; 2.62); *p* < 0.001	1.98 (1.48; 2.66); *p* < 0.001
Nutritional knowledge at a good or very good level	125 (75)	3.09 (1.90; 5.05); *p* < 0.001	3.34 (2.01; 5.54); *p* < 0.001	3.46 (2.07; 5.77); *p* < 0.001	67 (50)	0.32 (0.20; 0.53); *p* < 0.001	0.30 (0.18; 0.50); *p* < 0.001	0.29 (0.17; 0.48); *p* < 0.001	171 (44)	1.99 (1.49; 2.66); *p* < 0.001	1.96 (1.47; 2.62); *p* < 0.001	1.98 (1.48; 2.66); *p* < 0.001	122 (28)	0.50 (0.38; 0.67); *p* < 0.001	0.51 (0.38; 0.68); *p* < 0.001	0.50 (0.38; 0.68); *p* < 0.001
Nutritional knowledge at a very good level	47 (28)	6.27 (2.84; 13.86); *p* < 0.001	6.29 (2.77; 14.30); *p* < 0.001	6.21 (2.75; 14.04); *p* < 0.001	8 (6)	0.16 (0.07; 0.35); *p* < 0.001	0.16 (0.07; 0.36); *p* < 0.001	0.16 (0.07; 0.36); *p* < 0.001	28 (7)	2.16 (1.13; 4.10); *p* < 0.05	2.16 (1.13; 4.12); *p* < 0.05	2.23 (1.17; 4.26); *p* < 0.05	15 (3)	0.46 (0.24; 0.88); *p* < 0.05	0.46 (0.24; 0.88); *p* < 0.05	0.45 (0.23; 0.86); *p* < 0.05
Being a smoker	8 (5)	0.35 (0.15; 0.84); *p* < 0.05	0.32 (0.13; 0.79); *p* < 0.05	0.34 (0.14; 0.84); *p* < 0.05	17 (13)	2.85 (1.18; 6.84); *p* < 0.05	3.10 (1.26; 7.60); *p* < 0.05	2.93 (1.19; 7.23); *p* < 0.05	55 (14)	0.63 (0.44; 0.92); *p* < 0.05	0.65 (0.45; 0.93); *p* < 0.05	0.62 (0.43; 0.91); *p* < 0.05	89 (21)	1.58 (1.09; 2.28); *p* < 0.05	1.55 (1.07; 2.24); *p* < 0.05	1.60 (1.10; 2.32); *p* < 0.05
FS advice from the nutritionist	24 (14)	3.75 (1.36; 10.31); *p* < 0.05	3.91 (1.36; 11.24); *p* < 0.05	3.86 (1.34; 11.13); *p* < 0.05	5 (4)	0.27 (0.10; 0.73); *p* < 0.05	0.26 (0.09; 0.74); *p* < 0.05	0.26 (0.09; 0.75); *p* < 0.05	36 (9)	3.13 (1.58; 6.21); *p* < 0.01	3.12 (1.57; 6.18); *p* < 0.01	3.24 (1.62; 6.45); *p* < 0.001	12 (3)	0.32 (0.16; 0.63); *p* < 0.01	0.32 (1.16; 0.64); *p* < 0.01	0.31 (0.16; 0.62); *p* < 0.001
FS advice from the trainer	5 (3)	-	-	-	0 (0)	-	-	-	17 (4)	17.08 (2.24; 130.04); *p* < 0.01	17.08 (2.24; 130.26); *p* < 0.01	17.93 (2.35; 136.98); *p* < 0.01	1 (0)	0.06 (0.01; 0.45); *p* < 0.01	0.06 (0.01; 0.45); *p* < 0.01	0.06 (0.01; 0.43); *p* < 0.01
FS advice—other origins	57 (34)	0.53 (0.31; 0.93); *p* < 0.05	0.54 (0.31; 0.96); *p* < 0.05	0.52 (0.29; 0.93); *p* < 0.05	52 (39)	1.88 (1.08; 3.27); *p < 0.05*	1.85 (1.04; 3.27); *p* < 0.05	1.92 (1.08; 3.43); *p* < 0.05	56 (14)	0.56 (0.37; 0.84); *p* < 0.01	0.56 (0.37; 0.84); *p* < 0.01	0.54 (0.36; 0.82); *p* < 0.01	79 (18)	1.79 (1.19; 2.69); *p* < 0.01	1.78 (1.18; 2.68); *p* < 0.01	1.84 (1.22; 2.79); *p* < 0.01
Being a female	142 (86)	1.28 (0.69; 2.38); *p* = 0.434	1.07 (0.57; 2.03); *p* = 0.833	1.04 (0.54; 1.98); *p* = 0.908	111 (82)	0.78 (0.42; 1.45); *p* = 0.435	0.93 (0.49; 1.77); *p* = 0.834	0.96 (0.51; 1.83); *p* = 0.907	283 (73)	0.65 (0.47; 0.91); *p* < 0.05	0.60 (0.43; 0.84); *p* < 0.01	0.61 (0.43; 0.85); *p* < 0.01	347 (80)	1.53 (1.10; 2.12); *p* < 0.05	1.66 (1.19; 2.33); *p* < 0.01	1.65 (1.18; 2.31); *p* < 0.01
Being a male	24 (14)	0.78 (0.42; 1.45); *p* = 0.434	0.93 (0.49; 1.77); *p* = 0.833	0.96 (0.51; 1.83); *p* = 0.907	24 (18)	1.28 (0.69; 2.38); *p* = 0.435	1.07 (0.57; 2.03); *p* = 0.834	1.04 (0.55; 1.98); *p* = 0.907	103 (27)	1.49 (1.07; 2.07); *p* < 0.05	1.62 (1.16; 2.28); *p* < 0.01	1.61 (1.15; 2.25); *p* < 0.01	84 (19)	0.67 (0.48; 0.93); *p* < 0.05	0.62 (0.44; 0.86); *p* < 0.01	0.62 (0.44; 0.87); *p* < 0.01

^1^ PSHD—professionals, supplement users with healthy diet; ^2^ PnSLHD—professional non-supplement users with less healthy diet; **^3^** nPSHD—non-professional supplement users with healthy diet; ^4^ nPnSLHD—non-professionals, non-supplement users with less healthy diet; ^5^ BMI—body mass index.

## Data Availability

The data presented in this study are available on request from the corresponding author. The data are not publicly available due to privacy restrictions.

## Supplementary Materials

The following supporting information can be downloaded at: https://www.mdpi.com/article/10.3390/nu17172802/s1, Table S1: Supplementary data for variables (data source and assessment methods).

## Appendix A

**Table A1:** Characteristics of the study sample by identified clusters.

Variables	HP ^1^				nHP ^2^			
	Total Sample	PSHD ^3^	PnSLHD ^4^	*p*	Total Sample	nPSHD ^5^	nPnSLHD ^6^	*p*
Place of residence *n* (%)								
Village	68 (22.6)	40 (58.8)	28 (41.2)	NS Chi^2^ = 2.8	298 (36.3)	130 (33.4)	168 (38.9)	NS Chi^2^ = 4.3
City < 20,000 inhabitants	45 (15.0)	26 (57.8)	19 (42.2)		84 (10.2)	42 (10.8)	42 (9.7)	
City (20,000–100,000) inhabitants	85 (28.2)	50 (58.8)	35 (41.2)		216 (26.3)	100 (25.7)	116 (26.9)	
City > 100,000 inhabitants	103 (34.2)	50 (48.5)	53 (51.5)		223 (27.2)	117 (30.1)	106 (24.5)	
Number of residents in the house *n* (%)								
1 and 2	107 (35.6)	60 (36.1)	47 (34.8)	NS Chi^2^ = 0.6	189 (23.0)	103 (26.5)	86 (19.9)	NS Chi^2^ = 5.4
3 and 4	165 (54.8)	92 (55.4)	73 (54.1)		535 (65.2)	245 (63.0)	290 (67.1)	
≥5	29 (9.6)	14 (8.5)	15 (11.1)		97 (11.8)	41 (10.5)	56 (13.0)	
Educational level *n* (%)								
Primary School	4 (1.3)	1 (0.6)	3 (2.2)	NS Chi^2^ = 1.7	60 (7.3)	24 (6.2)	36 (8.3)	NS Chi^2^ = 5.9
High School	2 (0.7)	1 (0.6)	1 (0.7)		273 (33.3)	125 (32.1)	148 (34.3)	
University	277 (92.0)	153 (92.2)	124 (91.9)		456 (55.5)	219 (56.3)	237 (54.9)	
PhD	18 (6.0)	11 (6.6)	7 (5.2)		32 (3.9)	21 (5.4)	11 (2.5)	
Employment—full-time *n* (%)								
Other	15 (5.0)	10 (6.0)	5 (3.7)	NS Chi^2^ = 0.8	472 (57.5)	208 (53.5)	264 (61.1)	*p* < 0.05 Chi^2^ = 4.9
Full-time job	286 (95.0)	156 (94.0)	130 (96.3)		349 (42.5)	181 (46.5)	168 (38.9)	
Profession nutritionist *n* (%)								
Nutritionist	52 (17.3)	48 (28.9)	4 (3.0)	*p* < 0.001 Chi^2^ = 35.1	10 (1.2)	6 (1.5)	4 (0.9)	NS Chi^2^ = 0.6
Other	249 (82.7)	118 (71.1)	131 (97.0)		811 (98.8)	383 (98.5)	428 (99.1)	
Financial situation *n* (%)								
Low class	18 (6.0)	9 (5.4)	9 (6.7)	NS Chi^2^ = 1.8	91 (11.1)	34 (8.7)	57 (13.2)	NS Chi^2^ = 4.1
Middle class	270 (89.7)	152 (91.6)	118 (87.4)		716 (87.2)	348 (89.5)	368 (85.2)	
High class	13 (4.3)	5 (3.0)	8 (5.9)		14 (1.7)	7 (1.8)	7 (1.6)	
Quality of life								
(Mean ± SD) (min–max)	3.4 ± 0.8 (1.0–5.0)	3.4 ± 0.8 (1.0–5.0)	3.5 ± 0.9 (1.0–5.0)	NS	3.3 ± 0.8 (1.0–5.0)	3.4 ± 0.8 (1.0–5.0)	3.2 ± 0.9 (1.0–5.0)	*p* < 0.001
*n* (%)								
Modestly poor	2 (0.7)	1 (0.6)	1 (0.8)	NS Chi^2^ = 3.3	9 (1.1)	2 (0.5)	7 (1.6)	*p* < 0.01 Chi^2^ = 13.6
Modestly	32 (10.6)	17 (10.2)	15 (11.1)		115 (14.0)	40 (10.3)	75 (17.4)	
Normally	133 (44.2)	81 (48.9)	52 (38.5)		382 (46.5)	181 (46.5)	201 (46.5)	
Relatively comfortably	103 (34.2)	51 (30.7)	52 (38.5)		251 (30.6)	130 (33.4)	121 (28.0)	
Very comfortably	31 (10.3)	16 (9.6)	15 (11.1)		64 (7.8)	36 (9.3)	28 (6.5)	
SES ^7^—quartiles *n* (%)								
Low	67 (22.3)	38 (22.9)	29 (21.5)	*p* < 0.01 Chi^2^ = 11.8	296 (36.1)	118 (30.3)	178 (41.2)	*p* < 0.01 Chi^2^ = 11.0
Average	128 (42.5)	83 (50.0)	45 (33.3)		269 (32.8)	143 (36.8)	126 (29.2)	
High	106 (35.2)	45 (27.1)	61 (45.2)		256 (31.1)	128 (32.9)	128 (29.6)	
SES ^7^—(Mean ± SD) (min–max)	2.6 ± 1.1 (1.0–4.0)	2.5 ± 1.0 (1.0–4.0)	2.8 ± 1.1 (1.0–4.0)	*p* < 0.05	2.4 ± 1.2 (1.0–4.0)	2.5 ± 1.1 (1.0–4.0)	2.3 ± 1.2 (1.0–4.0)	*p* < 0.05
Smoking currently *n* (%)								
Yes	52 (17.3)	8 (4.8)	17 (12.6)	*p* < 0.05 Chi^2^ = 5.9	144 (17.5)	55 (14.1)	89 (20.6)	*p* < 0.05 Chi^2^ = 5.9
No	276 (91.7)	158 (95.2)	118 (87.4)		677 (82.5)	334 (85.9)	343 (79.4)	
BMI ^8^ (kg/m^2^) (Mean ± SD) (min–max)	23.0 ± 3.7 (17.0–41.0)	22.2 ± 3.2 (16.9–39.4)	23.9 ± 4.0 (18.0–41.0)	*p* < 0.001	23.3 ± 3.7 (14.1–40.1)	23.1 ± 3.1 (14.1–33.3)	23.6 ± 4.2 (16.2–40.1)	NS
*n* (%)								
Underweight (<18.5)	11 (3.6)	8 (4.8)	3 (2.2)	*p* < 0.001 Chi^2^ = 14.9	44 (5.4)	11 (2.8)	33 (7.6)	*p* < 0.001 Chi^2^ = 24.1
Normal (18.5–24.99)	218 (72.4)	130 78.3)	88 (65.2)		555 (67.6)	291 (74.8)	264 (61.1)	
Overweight (25–30)	55 (18.3)	25 (15.1)	30 (22.2)		171 (20.8)	73 (18.8)	98 (22.7)	
Obese (≥30)	17 (5.7)	3 (1.8)	14 (10.4)		51 (6.2)	14 (3.6)	37 (8.6)	
Knowledge concerning food supplements *n* (%)								
Incorrect	235 (78.1)	129 (77.7)	106 (78.5)	NS Chi^2^ = 0.03	605 (73.7)	291 (74.8)	214 (72.7)	NS Chi^2^ = 0.5
Correct	66 (21.9)	37 (22.3)	29 (21.5)		216 (26.3)	98 (25.2)	118 (27.3)	
The presence of chronic illness *n* (%)								
Yes	88 (29.2)	50 (30.1)	38 (28.1)	NS Chi^2^ = 0.7	241 (29.4)	109 (28.0)	132 (30.6)	NS Chi^2^ = 0.6
No	213 (70.8)	116 (69.9)	97 (71.9)		580 (70.6)	280 (72.0)	300 (69.4)	
Intake medications chronically *n* (%)								
Yes	87 (28.9)	51 (30.7)	36 (26.7)	NS Chi^2^ = 0.6	209 (25.5)	102 (26.2)	107 (24.8)	NS Chi^2^ = 0.2
No	214 (71.1)	115 (69.3)	99 (73.3)		612 (74.5)	287 (73.8)	325 (75.2)	
Food supplements use in the last 12 months *n* (%)								
Yes	182 (60.5)	110 (66.3)	72 (53.3)	NS Chi^2^ = 5.2	344 (41.9)	193 (49.6)	151 (35.0)	*p* < 0.001 Chi^2^ = 18.1
No	119 (39.5)	56 (33.7)	63 (46.7)		477 (58.1)	196 (50.4)	281 (65.0)	
Regular meals *n* (%)								
Yes	126 (41.9)	82 (49.4)	44 (32.6)	*p* < 0.05 Chi^2^ = 8.7	293 (35.7)	151 (38.8)	142 (32.9)	*p* < 0.01 Chi^2^ = 9.8
No	35 (11.6)	16 (9.6)	19 (14.1)		116 (14.1)	40 (10.3)	76 (17.6)	
Yes, but only few	140 (46.5)	68 (41.0)	72 (53.3)		412 (50.2)	198 (50.9)	214 (49.5)	
Number of meals								
(Mean ± SD) (min–max)	3.9 ± 0.9 (1.0–5.0)	4.5 ± 0.6 (3.0–5.0)	3.1 ± 0.7 (1.0–4.0)	*p* < 0.001	3.5 ± 0.9 (1.0–5.0)	3.7 ± 0.9 (1.0–5.0)	3.3 ± 0.9 (1.0–5.0)	*p* < 0.001
*n* (%)								
1	2 (0.7)	0	2 (1.5)	*p* < 0.001 Chi^2^ = 161.7	5 (0.6)	1 (0.3)	4 (0.9)	*p* < 0.001 Chi^2^ = 49.7
2	15 (5.0)	0	15 (11.1)		88 (10.7)	26 (6.7)	62 (14.4)	
3	93 (30.9)	13 (7.8)	80 (59.3)		326 (39.7)	125 (32.1)	201 (46.5)	
4	98 (32.6)	60 (36.1)	38 (28.1)		283 (34.5)	157 (40.4)	126 (29.2)	
5 or more	93 (30.9)	93 (56.0)	0		119 (14.5)	80 (20.6)	39 (9.0)	
Number of meals *n* (%)								
5 meals	93 (30.9)	93 (56.0)	0	*p* < 0.001 Chi^2^ = 109.4	119 (14.5)	80 (20.6)	39 (9.0)	*p* < 0.001 Chi^2^ = 22.0
Other	208 (69.1)	73 (44.0)	135 (100.0)		702 (85.5)	309 (79.4)	393 (91.0)	
Snacking between meals Mean ± SD (min–max)	1.0 ± 0.7 (1.0–2.0)	1.1 ± 0.8 (0.0–2.0)	0.8 ± 0.7 (0.0–2.0)	*p* < 0.001	0.9 ± 0.7 (0.0–2.0)	0.9 ± 0.7 (0.0–2.0)	1.0 ± 0.7 (0.0–2.0)	NS
Engagement in sport *n* (%)								
Yes	176 (58.5)	100 (60.2)	76 (56.3)	NS Chi^2^ = 0.5	402 (49.0)	376 (96.7)	26 (6.0)	*p* < 0.001 Chi^2^ = 672.9
No	125 (41.5)	66 (39.8)	59 (43.7)		419 (51.0)	13 (3.3)	406 (94.0)	
Diet quality index DQI ^9^								
(Mean ± SD) (min–max)	25.5 ± 9.1 (−0.4–69.6)	29.5 ± 8.2 (9.5–69.6)	20.7 ± 7.6 (−0.4–43.0)	*p* < 0.001	21.4 ± 9.6 (−14.4–49.1)	23.7 ± 9.1 (−0.4–49.1)	19.4 ± 9.5 (−14.4–45.8)	*p* < 0.001
Non-healthy diet index nHDI ^10^ (Mean ± SD) (min–max)	6.8 ± 4.6 (0–33.7)	6.8 ± 4.3 (1.0–28.4)	6.9 ± 5.1 (0–33.7)	NS	7.7 ± 5.2 (0.1–44.5)	6.4 ± 4.0 (0.2–24.0)	8.8 ± 5.8 (0.1–45.5)	*p* < 0.001
Pro-healthy diet index pHDI ^11^ (Mean ± SD) (min–max)	32.4 ± 8.9 (9.0–73.8)	36.3 ± 8.3 (14.9–73.8)	27.6 ± 7.1 (9.0–52.2)	*p* < 0.001	29.1 ± 9.5 (3.2–62.7)	30.1 ± 9.7 (6.6–62.7)	28.2 ± 9.2 (3.2–56.1)	*p* < 0.01
Sweet index SI ^12^								
(Mean ± SD) (min–max)	6.0 ± 5.8 (0.0–46.2)	6.0 ± 5.3 (0.5–28.8)	6.0 ± 6.4 (0.0–46.2)	NS	6.7 ± 6.4 (0.0–50.2)	5.2 ± 4.2 (0.0–25.0)	8.0 ± 7.6 (0.0–50.2)	*p* < 0.001
Recommendation to take food supplements in the last 12 months *n* (%)								
Yes	176 (58.5)	106 (63.9)	70 (51.9)	NS Chi^2^ = 4.7	89 (10.8)	44 (11.3)	45 (10.4)	NS Chi^2^ = 0.5
No	115 (38.2)	56 (33.7)	59 (43.7)		693 (84.4)	325 (83.6)	368 (85.2)	
Not sure	10 (3.3)	4 (2.4)	6 (4.4)		39 (4.8)	20 (5.1)	19 (4.4)	
Place of purchase of food supplements *n* (%)								
Pharmacy	177 (81.9)	104 (80.6)	73 (83.9)	NS Chi^2^ = 0.4	342 (74.8)	165 (69.9)	177 (80.1)	*p* < 0.05 Chi^2^ = 6.3
Others	39 (18.1)	25 (19.4)	14 (16.1)		115 (25.2)	71 (30.1)	44 (19.9)	
Sleep habits *n* (%)								
≤6 h	87 (28.9)	47 (28.3)	40 (29.6)	NS Chi^2^ = 0.6	212 (25.8)	95 (24.4)	117 (27.1)	NS Chi^2^ = 0.4
>7 h	214 (71.1)	71.7 (119.0)	70.4 (95.0)		609 (74.2)	294 (75.6)	315 (72.9)	
Food supplements recommendation *n* (%)								
Healthcare professionals	101 (46.8)	66 (51.2)	49 (56.3)	NS Chi^2^ = 0.6	254 (55.7)	125 (53.0)	129 (58.6)	NS Chi^2^ = 1.4
Others	115 (53.2)	63 (48.8)	38 (43.7)		202 (44.3)	91 (41.4)	111 (47.0)	
Frequency of consumption								
Carbonated or non-carbonated drinks (Coca-Cola, Fanta, Sprite) Mean ± SD (min–max)	0.1 ± 0.2 (0.0–2.0)	0.1 ± 0.2 (0.0–1.0)	0.2 ± 0.3 (0.0–2.0)	*p* < 0.05	0.2 ± 0.3 (0.0–2.0)	0.1 ± 0.2 (0.0–2.0)	0.2 ± 0.4 (0.0–2.0)	*p* < 0.001
Energy drink Mean ± SD (min–max)	0.01 ± 0.06 (0.0–1.0)	0.004 ± 0.039 (0.0–0.5)	0.01 ± 0.09 (0.0–1.0)	NS	0.02 ± 0.09 (0.0–1.0)	0.02 ± 0.10 (0.0–1.0)	0.01 ± 0.08 (0.0–1.0)	NS
Water Mean ± SD (min–max)	1.95 ± 0.25 (0.0–2.0)	1.98 ± 0.15 (1.0–2.0)	1.92 ± 0.34 (0.0–2.0)	NS	1.93 ± 0.31 (0.0–2.0)	1.96 ± 0.24 (0.0–2.0)	1.89 ± 0.36 (0.0–2.0)	*p* < 0.01
White bread and bakery products (wheat bread) Mean ± SD (min–max)	0.57 ± 0.56 (0.0–2.0)	0.633 ± 0.630 (0.0–2.0)	0.489 ± 0.438 (0.0–2.0)	*p* < 0.05	0.60 ± 0.583 (0.0–2.0)	0.490 ± 0.539 (0.0–2.0)	0.698 ± 0.604 (0.0–2.0)	*p* < 0.001
Dark bread (whole meal/rye dark) Mean ± SD (min–max)	0.6 ± 0.5 (0.0–2.0)	0.75 ± 0.59 (0.0–2.0)	0.41 ± 0.40 (0.0–2.0)	*p* < 0.001	0.42 ± 0.48 (0.0–2.0)	0.49 ± 0.49 (0.0–2.0)	0.36 ± 0.46 (0.0–2.0)	*p* < 0.001
White rice, pasta Mean ± SD (min–max)	0.7 ± 0.5 (0.0–2.0)	0.8 ± 0.6 (0.0–2.0)	0.6 ± 0.5 (0.0–2.0)	*p* < 0.01	0.7 ± 0.6 (0.0–2.0)	0.7 ± 0.6 (0.0–2.0)	0.8 ± 0.6 (0.0–2.0)	NS
Whole-grain pasta and rice Mean ± SD (min–max)	0.3 ± 0.4 (0.0–2.0)	0.3 ± 0.4 (0.0–2.0)	0.2 ± 0.3 (0.0–2.0)	*p* < 0.01	0.2 ± 0.4 (0.0–2.0)	0.3 ± 0.4 (0.0–2.0)	0.2 ± 0.3 (0.0–2.0)	*p* < 0.05
Fast food (French fries, burgers, pizza, hot dogs) Mean ± SD (min–max)	0.09 ± 0.10 (0.0–1.0)	0.08 ± 0.07 (0.0–0.5)	0.10 ± 0.12 (0.0–1.0)	NS	0.10 ± 0.11 (0.0–1.0)	0.09 ± 0.10 (0.0–0.5)	0.10 ± 0.12 (0.0–1.0)	*p* < 0.05
Fried foods (meat or flour-based foods, such as dumplings and pancakes) Mean ± SD (min–max)	0.09 ± 0.13 (0.0–1.0)	0.09 ± 0.12 (0.0–1.0)	0.10 ± 0.15 (0.0–1.0)	NS	0.14 ± 0.19 (0.0–2.0)	0.11 ± 0.15 (0.0–1.0)	0.16 ± 0.22 (0.0–2.0)	*p* < 0.001
Butter to spread on bread or add to meals/for frying/for baking Mean ± SD (min–max)	0.42 ± 0.46 (0.0–2.0)	0.41 ± 0.49 (0.0–2.0)	0.44 ± 0.43 (0.0–2.0)	NS	0.41 ± 0.47 (0.0–2.0)	0.32 ± 0.42 (0.0–2.0)	0.49 ± 0.50 (0.0–2.0)	*p* < 0.001
Lard to spread on bread, or as a complement to meals/for frying/for roasting Mean ± SD (min–max)	0.02 ± 0.15 (0.0–2.0)	0.03 ± 0.18 (0.0–2.0)	0.02 ± 0.10 (0.0–1.0)	NS	0.03 ± 0.16 (0.0–2.0)	0.02 ± 0.11 (0.0–1.0)	0.04 ± 0.20 (0.0–2.0)	NS
Vegetable oils or margarines or mixtures of butter and margarines Mean ± SD (min–max)	0.12 ± 0.27 (0.0–2.0)	0.10 ± 0.24 (0.0–2.0)	0.14 ± 0.31 (0.0–2.0)	NS	0.21 ± 0.36 (0.0–2.0)	0.18 ± 0.35 (0.0–2.0)	0.23 ± 0.37 (0.0–2.0)	*p* < 0.05
Milk (including flavoured milk. hot chocolate, latte) Mean ± SD (min–max)	0.59 ± 0.64 (0.0–2.0)	0.71 ± 0.70 (0.0–2.0)	0.44 ± 0.53 (0.0–2.0)	*p* < 0.001	0.51 ± 0.57 (0.0–2.0)	0.45 ± 0.54 (0.0–2.0)	0.56 ± 0.59 (0.0–2.0)	*p* < 0.01
Fermented dairy Mean ± SD (min–max)	0.72 ± 0.62 (0.0–2.0)	0.91 ± 0.66 (0.0–2.0)	0.5 ± 0.47 (0.0–2.0)	*p* < 0.001	0.51 ± 0.51 (0.0–2.0)	0.56 ± 0.53 (0.0–2.0)	0.48 ± 0.49 (0.0–2.0)	*p* < 0.05
Fresh cheese curd products (cottage cheese) Mean ± SD (min–max)	0.28 ± 0.42 (0.0–2.0)	0.36 ± 0.51 (0.0–2.0)	0.17 ± 0.23 (0.0–1.0)	*p* < 0.001	0.19 ± 0.32 (0.0–2.0)	0.23 ± 0.37 (0.0–2.0)	0.16 ± 0.29 (0.0–2.0)	*p* < 0.01
Cheese (including processed cheese) Mean ± SD (min–max)	0.54 ± 0.57 (0.0–2.0)	0.65 ± 0.61 (0.0–2.0)	0.41 ± 0.46 (0.0–2.0)	*p* < 0.001	0.4 ± 0.46 (0.0–2.0)	0.4 ± 0.46 (0.0–2.0)	0.4 ± 0.46 (0.0–2.0)	NS
Cured meats, sausages Mean ± SD (min–max)	0.11 ± 0.17 (0.0–1.0)	0.10 ± 0.14 (0.0–1.0)	0.13 ± 0.19 (0.0–1.0)	NS	0.16 ± 0.24 (0.0–2.0)	0.14 ± 0.20 (0.0–2.0)	0.18 ± 0.28 (0.0–2.0)	*p* < 0.05
Red meat (pork, beef, veal, lamb) Mean ± SD (min–max)	0.33 ± 0.29 (0.0–2.0)	0.33 ± 0.27 (0.0–2.0)	0.34 ± 0.32 (0.0–2.0)	NS	0.37 ± 0.36 (0.0–2.0)	0.36 ± 0.36 (0.0–2.0)	0.39 ± 0.36 (0.0–2.0)	NS
White meat (turkey, chicken) Mean ± SD (min–max)	0.53 ± 0.27 (0.0–2.0)	0.56 ± 0.28 (0.0–2.0)	0.50 ± 0.24 (0.1–2.0)	NS	0.61 ± 0.43 (0.0–2.0)	0.64 ± 0.46 (0.0–2.0)	0.58 ± 0.41 (0.0–2.0)	NS
Fish, shellfish Mean ± SD (min–max)	0.49 ± 0.31 (0.0–2.0)	0.54 ± 0.31 (0.0–2.0)	0.42 ± 0.3 (0.1–2.0)	*p* < 0.01	0.45 ± 0.32 (0.0–2.0)	0.49 ± 0.34 (0.0–2.0)	0.42 ± 0.30 (0.0–2.0)	*p* < 0.01
Eggs Mean ± SD (min–max)	0.51 ± 0.33 (0.0–2.0)	0.54 ± 0.31 (0.1–2.0)	0.47 ± 0.35 (0.0–2.0)	NS	0.52 ± 0.4 (0.0–2.0)	0.57 ± 0.42 (0.0–2.0)	0.48 ± 0.38 (0.0–2.0)	*p* < 0.001
Legume-based foods, e.g., beans, peas, soybeans, lentils Mean ± SD (min–max)	0.45 ± 0.42 (0.0–2.0)	0.52 ± 0.46 (0.1–2.0)	0.36 ± 0.46 (0.0–2.0)	*p* < 0.01	0.44 ± 0.43 (0.0–2.0)	0.49 ± 0.49 (0.0–2.0)	0.40 ± 0.36 (0.0–2.0)	*p* < 0.01
Potatoes (excluding French fries) Mean ± SD (min–max)	0.34 ± 0.30 (0.0–2.0)	0.39 ± 0.32 (0.0–2.0)	0.27 ± 0.26 (0.0–2.0)	*p* < 0.001	0.32 ± 0.29 (0.0–2.0)	0.32 ± 0.3 (0.0–2.0)	0.32 ± 0.29 (0.0–2.0)	NS
Fruits Mean ± SD (min–max)	1.6 ± 0.6 (0.0–2.0)	1.7 ± 0.5 (0.0–2.0)	1.3 ± 0.7 (0.0–2.0)	*p* < 0.001	1.2 ± 0.7 (0.0–2.0)	1.3 ± 0.7 (0.0–2.0)	1.1 ± 0.7 (0.0–2.0)	*p* < 0.001
Vegetables Mean ± SD (min–max)	1.5 ± 0.7 (0.0–2.0)	1.7 ± 0.6 (0.1–2.0)	1.3 ± 0.7 (0.0–2.0)	*p* < 0.001	1.1 ± 0.7 (0.0–2.0)	1.2 ± 0.7 (0.0–2.0)	1.0 ± 0.7 (0.0–2.0)	*p* < 0.001
Sweets (confectionery, biscuits, cakes, chocolate bars, cereal bars) Mean ± SD (min–max)	0.3 ± 0.4 (0.0–2.0)	0.3 ± 0.3 (0.0–2.0)	0.3 ± 0.4 (0.0–2.0)	NS	0.3 ± 0.4 (0.0–2.0)	0.3 ± 0.3 (0.0–2.0)	0.4 ± 0.4 (0.0–2.0)	*p* < 0.001
Instant soups or ready-made soups Mean ± SD (min–max)	0.2 ± 0.4 (0.0–2.0)	0.20 ± 0.46 (0.0–2.0)	0.20 ± 0.41 (0.0–2.0)	NS	0.25 ± 0.45 (0.0–2.0)	0.26 ± 0.48 (0.0–2.0)	0.24 ± 0.42 (0.0–2.0)	NS
Canned meats Mean ± SD (min–max)	0.01 ± 0.05 (0.0–0.5)	0.01 ± 0.03 (0.0–0.14)	0.01 ± 0.06 (0.0–0.50)	NS	0.03 ± 0.11 (0.0–2.0)	0.02 ± 0.12 (0.0–2.0)	0.03 ± 0.10 (0.0–1.0)	NS
Vegetables (jar), e.g., pickled vegetables, tinned Mean ± SD (min–max)	0.05 ± 0.10 (0.0–0.5)	0.04 ± 0.09 (0.0–0.5)	0.05 ± 0.11 (0.0–0.5)	NS	0.07 ± 0.17 (0.0–2.0)	0.08 ± 0.19 (0.0–2.0)	0.07 ± 0.15 (0.0–1.0)	NS
Fruit juice Mean ± SD (min–max)	0.17 ± 0.30 (0.0–2.0)	0.18 ± 0.34 (0.0–2.0)	0.15 ± 0.25 (0.0–2.0)	NS	0.23 ± 0.38 (0.0–2.0)	0.21 ± 0.40 (0.0–2.0)	0.25 ± 0.36 (0.0–2.0)	NS
Vegetable juices or fruit and vegetable juices Mean ± SD (min–max)	0.1 ± 0.28 (0.0–2.0)	0.13 ± 0.36 (0.0–2.0)	0.05 ± 0.11 (0.0–0.5)	*p* < 0.05	0.11 ± 0.28 (0.0–2.0)	0.13 ± 0.32 (0.0–2.0)	0.11 ± 0.23 (0.0–2.0)	NS
Alcoholic beverage Mean ± SD (min–max)	0.12 ± 0.17 (0.0–1.0)	0.1 ± 0.15 (0.0–1.0)	0.14 ± 0.19 (0.0–1.0)	*p* < 0.05	0.18 ± 0.28 (0.0–2.0)	0.18 ± 0.27 (0.0–2.0)	0.18 ± 0.30 (0.0–2.0)	NS

^1^ HP—healthcare professionals; ^2^ nHP—non-healthcare professionals; ^3^ PSHD—professional supplement users with healthy diet; ^4^ PnSLHD^—^professional non-supplement users with less healthy diet; **^5^** nPSHD—non-professional supplement users with healthy diet; ^6^ nPnSLHD—non-professional non-supplement users with less healthy diet; ^7^ SES—socioeconomic status; ^8^ BMI—body mass index; ^9^ DQI—diet quality index; ^10^ nHDI^—^non-healthy diet index included intake products: carbonated or non-carbonated drinks, energy drinks, fast food, fried foods, butter, lard, vegetable oils or margarines, processed cheese, cured meats and sausages, sweets, instant soups, canned meats, canned vegetables, candy, cookies, cakes, chocolate bars, muesli/granola bars, donuts, chocolate, ice cream, sweetened yogurt, cakes, gums, jelly, honey, alcoholic beverages; ^11^ pHDI—pro-healthy diet index included intake products: water, white bread, dark bread, white rice and pasta, whole-grain pasta and rice, milk, fermented dairy products, fresh cheese curd, red meat, white meat, fish and shellfish, eggs, legume, potatoes, fruits, vegetables, fruit juice, vegetable juice; ^12^ SI—sweet index included intake candy, cookies, cakes, chocolate bars, muesli/granola bars, donuts, chocolate, ice cream, sweetened yogurt, cakes, gums, jelly, honey.
