# Supplementary material for: Exploring the Lifestyle and Dietary Patterns of Food Supplement and Non-Food Supplement Users: A Cross-Sectional Study in the Portuguese Population
Source: Nutrients. 2025 Aug 28;17(17):2802. doi: 10.3390/nu17172802 (PMC12430018; doi:10.3390/nu17172802)
Supplement: Supplementary file 1 [file nutrients-17-02802-s001.zip › nutrients-3821899-Table S1. Supplementary data for variables (data source and assessment methods) R1.pdf]

nutrients

MDPI

Table S1. Supplementary data for variables (data source and assessment methods)

Variables of interest	Data Sources	Assessment Methods
**The main questions for subpopulations**		
HP[1] Status	Self-reported information through survey question: "What's your profession?"	multiple choice question<br>Including medical doctor, pharmacist, nutritionist and dietitians, nurse, teacher, engineer, lawyer, jurist, factory worker, veterinary doctor, administrative, commercial, physiotherapist, diagnostic and therapeutic technician, professional athlete, professional coach, banking, manager, other professional therapies (e.g., acupuncture, osteopath, etc.), other.<br>HP status - medical doctor, pharmacist, nutritionist and dietitians, nurse, veterinary doctor, physiotherapist, diagnostic and therapeutic technician, other professional therapies (e.g., acupuncture, osteopath, etc.), other
FS[2] User	Self-reported information through survey question: "Have you taken any food supplements in the last 12 months?"	multiple choice question<br>Yes or No
**Additional questions for evaluation of attitudes through food supplements and health information**		
The presence of chronic illness	Self-reported information through survey question: Do you have any chronic illnesses?	multiple choice question<br>No or Yes with multiple options, with examples of chronic illness such as diabetes, depression, obesity, high cholesterol, etc<br>Categorisation:<br>No and all the "Yes answers"
Intake medications chronically	Self-reported information through survey question: "Do you take any medication chronically?"	multiple choice question<br>Yes or No
Knowledge about FS[2]	Self-reported information through survey question: "What are food supplements for you?"	multiple choice question:<br>a medicine<br>other health product; a conventional food; a food with some specificities

		Categorisation:<br>Correct answer: “a food with some specificities”<br>Incorrect answer: all the others options
Place of purchase of FS[2]	Self-reported information through survey question:<br>“Where do you buy food supplements?”	multiple choice question with the options:<br>Supermarket, Pharmacy, Internet, Dietetic center, “are offered”<br>Categorisation:<br>Pharmacy and all the other answers
Recommendation to take FS[2] in the last 12 months	Self-reported information through survey question:<br>Did you prescribe/advised any food supplement in the last 12 months?	multiple choice question<br>Yes, no or not sure
FS[2] recommendation	Self-reported information through survey question:<br>“Who recommended the food supplements to you?”	multiple choice question with the options:<br>colleagues, relatives, friends, medical doctor, trainer, nutritionist, others.<br>Categorisation:<br>[1]HP – nutritionist, medical doctor and pharmacist<br>Others - colleagues, relatives, friends, trainer and other
FS[2] advice from the nutritionist	Self-reported information through survey question:<br>“Who recommended the food supplements to you?”	multiple choice question with the options:<br>colleagues, relatives, friends, medical doctor, trainer, nutritionist, others.<br>Nutritionist – nutritionist
FS[2] advice from the trainer	Self-reported information through survey question:<br>“Who recommended the food supplements to you?”	multiple choice question with the options:<br>colleagues, relatives, friends, medical doctor, trainer, nutritionist, others.<br>Trainer – trainer
FS[2] advice - other origins	Self-reported information through survey question:<br>“Who recommended the food supplements to you?”	multiple choice question with the options:<br>colleagues, relatives, friends, medical doctor, trainer, nutritionist.<br>Other origins – others
Sleep habits	Self-reported information through survey question:<br>How many hours do you sleep a day?	multiple choice question with the options:<br>≤6h per day; 7h or 8h per day; ≥ 9h<br>Categorisation:<br>≤6h per day and ≥ 7h per day

[1]HP - Healthcare professionals; [2]FS - Food supplements.
